# Peri-procedural respiratory complications in patients undergoing pulmonary vein isolation under procedural sedation and analgesia: Incidence and predictive factors

**DOI:** 10.1016/j.ijcha.2025.101822

**Published:** 2025-10-14

**Authors:** Marloes Homberg, Konstanze Betz, Sander M.J. van Kuijk, Justin Luermans, E.A. Joosten, Wolfgang Buhre, Ulrich Schotten, Kevin Vernooy, Dominik Linz, Esther Bouman

**Affiliations:** a Department of Anaesthesiology, Maastricht University Medical Centre, Maastricht, the Netherlands; b Department of Cardiology, Cardiovascular Research Institute Maastricht (CARIM), Maastricht University Medical Centre, Maastricht, the Netherlands; c Department of Clinical Epidemiology and Medical Technology Assessment (KEMTA), Maastricht University Medical Centre, Maastricht, the Netherlands; d Department of Anaesthesiology, University Medical Centre Utrecht, Utrecht, the Netherlands; e Department of Biomedical Sciences, Faculty of Health and Medical Sciences, University of Copenhagen, Copenhagen, Denmark

**Keywords:** Procedural sedation and analgesia, Respiratory complications, Atrial fibrillation, Catheter ablation

## Abstract

**Background and Objectives:**

Catheter ablation (CA) in patients with atrial fibrillation (AF) can be performed under procedural sedation and analgesia (PSA). Risk factors for respiratory complications during PSA are unclear. We aimed to determine the incidence and severity of respiratory complications during PSA and identify predictive factors for development of per-procedural respiratory complications.

**Methods:**

Patients with AF receiving PSA in the MUMC^+^ for CA were included. Respiratory complications were defined as the need of an oral/nasal airway, a non-rebreathing mask, high flow oxygen, a hypoxemic event or conversion to general anaesthesia.

**Results:**

In total 232 procedures (42.2 % cryoballoon ablation; 57.3 % RF ablation) were performed. Most patients were male (62.1 %), with a mean age of 64 ± 9.3 (mean ± SD), an activity level ≥ 4 METS (94.8 %) and paroxysmal AF (68.5 %). Respiratory complications occurred in 42.2 %. A multiple logistic regression model including sedation duration, age > 50 years, neck circumference > 40 cm, visceral fat percentage, self-reported tiredness, apnoea hypopnoea index (AHI), medical background of diagnosed sleep apnoea (OSAS) and chronic obstructive pulmonary disease (COPD) identified patients at risk with an AUROC 0.72, 95 % CI: 0.65–0.78.

**Conclusion:**

In patients with AF undergoing CA under PSA, per-procedural respiratory complications occur in 42.2 % of the cases. Duration of the PSA, a neck circumference > 40 cm, visceral fat percentage, tiredness, AHI, OSAS and COPD may help to identify patients at risk for per-procedural respiratory complications. Although age > 50 years is a recognized risk factor, this cut-off had limited discriminative value in our cohort, as most patients were older than 50.

## Highlights

• Respiratory complications are common during deep sedation for atrial fibrillation ablation.
• Body composition is associated with the risk of respiratory complications.
• Improved pre-assessment may support pre-procedural risk stratification.

## 1 Introduction

Catheter ablation (CA) for atrial fibrillation (AF) has become one of the most common procedures in the cardio-electrophysiology lab [1]. In patients with AF, CA is performed either under procedural sedation and analgesia (PSA) or under general anaesthesia (GA) [[1], [2], [3]]. The worldwide European Heart Rhythm Association (EHRA) survey in 2021 showed that GA was the most commonly used anaesthetic technique (40.5 %), followed by conscious sedation (32.0 %) and deep sedation (27.5 %) [4]. Under deep sedation, the level of consciousness approaches that of general anaesthesia, and in most centres an anaesthesiologist, a second physician, or a specially trained nurse is required to be present [1,5,6]. In addition to alleviating pain and anxiety, an important goal for anaesthesia during AF ablation is to minimize patient movement as this improves catheter stability [4]. Therefore, general anaesthesia or deep sedation has frequently been preferred over conscious sedation [4].

Patient-related risk factors of perioperative respiratory complications include advanced age, chronic obstructive pulmonary disease (COPD), poorly controlled asthma, heart failure, poor systemic health status defined as an ASA classification of 2 or higher, cigarette smoking within 8 weeks before surgery, preoperative anaemia (haemoglobin concentration lower than 10 g/dL), low preoperative peripheral oxygen saturation (SpO_2_), functional dependence, patients with current respiratory tract infections, and obstructive sleep apnoea (OSA) [[7], [8], [9]]. Obesity, COPD and sleep-disordered breathing (SDB) are common concomitant conditions in AF-patients [10,11]. Although the STOP-BANG questionnaire is a broadly used screening tool for SDB, and therefore used during pre-assessment in the anaesthesia outpatient clinic, it is known not to be very accurate in patients with AF [12,13]. These conditions may affect the incidence of respiratory complications during PSA.

Interdisciplinary teamwork, patient selection, close communication, accurate planning, establishing protocols and pathways for help may facilitate safe, efficient procedural care and non-operating room anaesthesia (NORA) [3]. In NORA, the anaesthesia technique may range from minimal sedation to general anaesthesia. The occurrence of hypoxemia is one of the most common issues of sedation in non-intubated patients [14]. A considerable number of patients undergoing PSA for CA are at risk of hypoxemia during PSA [15]. Increased duration of medical procedures has shown a positive correlation with hypoxemia during PSA [15]. In patients undergoing PSA during procedures in the heart catheterization room, a 54 % incidence of hypoxemia was found [15]. However, patients with coexisting cardiovascular or pulmonary disease are more at risk for hypoxemic cardiac or cerebral events [7,8,16].

Nevertheless, the identification of patient-related factors, in particular in view of respiratory complications during moderate or deep sedation, is known to be very limited. In a multidisciplinary approach, involving electrophysiologists and anaesthesiologists, patient selection is needed to outweigh the risks and benefits related to PSA and GA. All the more important, as AF-ablation has increased enormously in recent years. And therefore the demand for anaesthesia support for AF ablations does not match the anaesthesia availability [1]. The primary aim of this study was to determine the incidence and severity of per-procedural respiratory complications during CA in patients with AF under PSA. The secondary aim of this study was to identify predictive factors for the development of per-procedural respiratory complications during RFCA in patients with AF under PSA. Ultimately, this knowledge could help optimize patient selection for PSA during CA.

## 2 Methods

### 2.1 Study design and ethical approval

This retrospective single-centre study was performed at the Maastricht University Medical Centre (MUMC^+^) in the Netherlands. This is a secondary data analysis of the ongoing ISOLATION cohort study (NCT04342312) and ISOLATION ‘light’ registry [17]. The ISOLATION cohort study and ISOLATION ‘light’ registry were approved by the ethical review boards MUMC^+^ (METC numbers 19–052, 2019 1022) and Radboud-UMC (METC number 2019–5629). This study was conducted in compliance with the Declaration of Helsinki and the Good Clinical Practice guidelines. All participants provided written informed consent.

### 2.2 Study population

Patient selection for PSA during CA is based on a combination of guidelines and practice. PSA is provided and monitored by anaesthesia care providers (ACPs) at MUMC^+^ since 2013. Known exclusion criteria for PSA during CA at MUMC^+^ are: Body Mass Index (BMI) > 32 kg/m^2^, Obstructive Sleep Apnoea (OSA) diagnosed or high risk based on STOP-Bang questionnaire, reflux and diaphragmatic hernia. Also a poor pulmonary condition, an electrophysiologist request for GA or a lack of informed consent for PSA is a reason for excluding patients from PSA. Patients with AF receiving anaesthesia in the MUMC^+^ for CA were included in this study between October 2020 and July 2023. Patients were excluded from this study if they received GA, PSA was performed but not registered into the patient data management system (PDMS), or the patient did not undergo the remote sleep test for SDB (the Virtual-SAFARI approach [18]).

Systematic screening for common comorbidities and triggers for AF is part of the ISOLATION study and is integrated in a standardized care pathway. Patients are screened for hypertension, obesity, hyperlipidaemia, diabetes mellitus, and chronic obstructive pulmonary disease (COPD) according to current AF guidelines [4]. The standard work-up for ablation in the participating centres consists a systematic collection of clinical information, vital signs, 12-lead ECG, blood tests, echocardiography, imaging to assess pulmonary vein anatomy (either computed tomography or cardiac magnetic resonance imaging), and pre- anaesthesia assessment by a physician assistant of the department of anaesthesiology. The pre-anaesthesia assessment is standard anaesthesia care. Previous medical records are reviewed, the patient is interviewed, a focused physical examination is performed, and available (laboratory) tests are reviewed. This patient evaluation contributes to the likelihood of satisfactory sedation and decreases the likelihood of adverse outcomes for moderate and deep sedation. Additionally, results of the following additional diagnostics are included in the ISOLATION study: determination of body composition, pre-procedural rhythm monitoring, extended surface electrocardiogram, biomarker testing, genetic analysis, and questionnaires [17].

### 2.3 Interventions and outcome measures

CA was always performed under sedation by an ACP. Types of CA were cryoballoon ablation or radiofrequency AF ablation. ACPs involved in moderate to deep sedation are sedation practitioners working under either direct or indirect supervision (direct support available within 5 min) from an anaesthesiologist. PSA included a combination of a continuous infusion with propofol in combination with a short-acting opioid like remifentanil or alfentanil. Para-acetylaminophenol was administered to prevent post-procedural pain if necessary in combination with piritramide or morphine. Patients were continuously observed. Non-invasive blood pressure, peripheral oxygen saturation (SpO_2_), a 5-lead electrocardiogram, bispectral index (BIS), respiratory rate and waveform capnography were monitored. ACPs assessed the depth of sedation periodically throughout the procedure by utilizing the Modified Ramsay Sedation Scale (RSS) [19,20]. Oxygen was administered with a flow of 2–5 L/min. In case of hypotension, a vasoactive drug was administered. In case of an upper airway obstruction, airway repositioning, tactile stimulation or airway (nasal or oral) was applied. In our centre, the use of a nasal or oral airway is not part of routine care. These devices are only inserted in response to clinically significant airway obstruction or desaturation, not as prophylactic use. The setting complies with international sedation guidelines [5,6].

After reaching an appropriate level of sedation, femoral venous access for CA was obtained using ultrasound guidance. A decapolar catheter was placed in the coronary sinus (Webster CS catheter, Biosense Webster, Inc, Diamond Bar, CA, United States). Transesophageal echocardiography-guided double *trans*-septal access was performed: the first access was realized using a SL-0 sheath (Abbott, Inc, Chicago, IL, United States) and a Brockenbrough technique. Intravenous heparin was administered after the *trans*-septal access, targeting an activated clotting time (ACT) > 300 s.

Catheter ablation by cryoballoon in described in detail elsewhere [21]. In brief, the transseptal sheath was exchanged over a guidewire for the 15F Flexcath Advance sheath and continuously flushed with heparinized saline and the cryoballoon catheter (Arctic Front™ cryoballoon, Medtronic, Inc.) was introduced into the left atrium and a spiral mapping catheter (20  mm diameter; Achieve, Medtronic, Inc.) was advanced into the target pulmonary veins to record electrical activity. A stable occlusion was verified by contrast dye injection. The minimum cryoballoon cut-off temperature was set at − 60 °C. A time-to-effect based ablation protocol was utilized for both cryoballoon systems. The procedural endpoint was the disappearance of PV signals verified via the circular mapping catheter after the freeze cycle.

Catheter ablation by radiofrequency in described in detail elsewhere [22]. In brief, a second *trans*-septal access was realized using a deflectable sheath (Vizigo sheath, Biosense Webster, Inc) and an over the wire technique. A high-density mapping catheter (OctaRay catheter, Biosense Webster, Inc) was used to perform an electro-anatomical reconstruction of the left atrium (LA) (CARTO 3 System Version 7, Biosense Webster, Inc). The ablation catheter (ThermoCool® SmartTouch® Surround Flow (STSF), Biosense Webster, Inc) was used for pulmonary vein isolation by a wide area circumferential ablation. Target range for CF was 10–25  g. Target ablation index was 500–550 using 45 Watts or 380–400 using 35 Watts for the anterior and posterior segments, respectively. The inter-lesion distance was set to 5–6  mm. Routine use of an oesophageal temperature probe is avoided in sedated patients due to its potential to cause *peri*-procedural respiratory complications.

After the procedure, a systematic *trans*-thoracic echocardiography was performed to rule out pericardial effusion. Figure-of-8 stitch and 4 h compressive bandaging was placed at the site of femoral puncture to avoid bleeding. Patients were then transferred to the recovery room for monitoring. After clinical observation on the short stay unit, patients were discharged on the same day in case of an uneventful procedure and recovery.

**Fig. 1:**
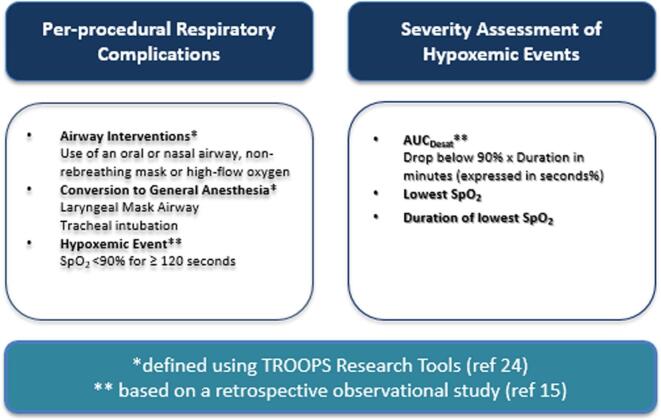
Descriptions of respiratory complications and the severity assessment of hypoxemic events.

The Tracking and Reporting Outcomes Of Procedural Sedation (TROOPS) comprehensive research tool [23] was used to define per-procedural respiratory complications during sedation. Events were grouped under the broader category of “respiratory complications,” this stratified classification allows for a more nuanced interpretation. Respiratory complications were defined as mild when transient hypoxaemia or airway obstruction was relieved by the use of a nasal airway, intermediate when relieved by an oral airway, a non-rebreathing mask or high-flow oxygen, and severe when a prolonged hypoxemic event occurred (peripheral oxygen saturation < 90 % lasting ≥ 120 s) or when airway obstruction necessitated conversion to general anaesthesia with a laryngeal mask airway (LMA) or tracheal intubation. The definition for a hypoxemic event was based on a retrospective observational study on hypoxemia during procedural sedation in adult patients [15]. To express the severity of respiratory complications, in addition to the incidence of hypoxemia, we determined the area under the curve (AUC_Desat_), the lowest mean saturation and the duration of the lowest saturation. For each hypoxemic event AUC_Desat_ (by multiplying duration in minutes and drop in saturation under 90 %) were determined in seconds % [24]. Fig. 1 shows the descriptions of respiratory complications and the severity assessment of hypoxemic events.

### 2.4 Clinical assessment

Vital signs were continuously monitored using the Philips Medical Systems Intellivue MX800 1&2 and automatically stored in a patient data management system (PDMS). Administrated medication during PSA and recovery stay, duration of sedation, and CA were entered into the PDMS. For this study, the prospective dataset from ISOLATION study and ISOLATION ‘light’ registry were completed with anaesthesiology data from PDMS and the pre-operative anaesthesia evaluation form. All data required for this study was extracted from the hospital electronic health systems and incorporated in the online database Castor EDC.

### 2.5 Statistical methods

Patients’ characteristics were described using descriptive statistics. The distribution of continuous variables was assessed using histograms. Depending on the distribution, we used the independent-samples *t*-test (in case of normal distribution) or the Mann-Whitney *U* test. Categorical and binary variables were tested using Pearson’s chi-squared test. The associations between patient- or procedure-related factors and respiratory complications were estimated using univariable and multivariable logistic regression. The alpha used for variable selection was set at 0.30, a rule often used for detection of risk factors or predictors. We used backward elimination with all factors associated with respiratory complications. The discriminative ability of patient- and procedure-related characteristics was evaluated by calculating the area under the receiver operating characteristics (ROC) curve (AUROC). An AUROC above 0.7 was considered to indicate a sufficient level of discrimination. Missing data were imputed using multiple imputations with fully conditional specification. Imputation was performed in SPSS version 28.0.0.0. Statistical analysis was performed using R version 4.1.3.

## 3 Results

### 3.1 Patient characteristics

We included 232 participants. Most sedations were performed in men (62.1 %). The mean (± SD) age was 64 ± 9.3, BMI 27.0 ± 3.0, with an activity level ≥ 4 METS (94.8 %) and paroxysmal AF (68.5 %). Most patients had an American Society of Anesthesiologists (ASA) classification II (84.1 %). A cryoballoon ablation was performed in 42.2 % and RF ablation in 57.3 % of the cases. Patient characteristics at baseline of all procedures, without respiratory complications group and with respiratory complications group are summarized in Table 1, with additional details provided in Supplemental Table 1.

**Table 1:** Baseline characteristics of all procedures, without respiratory complications group and with respiratory complications group. Values are number (percentages) unless stated otherwise.

**Characteristic**	**Total**	**No complications**	**Respiratory complications**
	n = 232	n = 134	n = 98
**Sex**			
Male	144 (62.1)	78 (58.2)	66 (67.3)
Female	88 (37.9)	56 (41.8)	32 (32.7)
**Age, years – mean (SD)**	64 (±9.3)	63 (±9.7)	64 (±8.7)
**BMI, kg/m^2^ – mean (SD)**	27.0 (±3.0)	26.4 (±3.0)	27.4 (±2.9)
**Activity level < 4 METS**	12 (5.2)	8 (6.0)	4 (4.1)

**ASA classification**			
I	2 (0.8)	1 (0.7)	1 (1.0)
II	195 (84.1)	115 (85.9)	80 (81.6)
III	35 (15.0)	18 (13.4)	17 (18.4)

**Type of AF**			
Paroxysmal	159 (68.5)	97 (72.4)	62 (63.3)
Persistent	73 (31.4)	37 (27.6)	36 (36.7)

**Type of ablation**			
Cryoballoon	98 (42.2)	64 (47.8)	34 (34.7)
Radiofrequency	133 (57.3)	69 (51.5)	64 (65.3)

**Comorbidities**			
CAD	19 (8.2)	9 (6.7)	10 (10.2)
CHF	25 (10.8)	15 (11.2)	10 (10.2)
Diabetes mellitus	15 (6.5)	7 (5.2)	8 (8.2)
OSAS	21 (9.1)	16 (11.9)	5 (5.1)
COPD	10 (4.3)	8 (6.0)	2 (2.0)

**Body composition**			
Fat percentage, mean (SD)	30.0 (±7.6)	29.7 (±1.9)	30.1 (±7.1)
Muscle percentage, mean (SD)	30.0 (±3.9)	30.4 (±4.2)	30.2 (±3.4)
Visceral fat percentage, mean (SD)	11.0 (±3.8)	10.7 (±4.0)	12.2 (±3.3)

**SDB indices**			
AHI, mean (SD)	13.4 (±10.5)	12.6 (±9.4)	14.5 (±11.7)
ODI, mean (SD)	5.6 (±6.1)	5.4 (±5.7)	6.5 (±6.6)
Desaturation nadir, % (SD)	91.4 (±6.2)	91.8 (±1.3)	91.7 (±1.4)
Saturation < 90 %, min (SD)	2.2 (±7.0)	2.1 (±7.1)	2.3 (±6.9)

**STOP-BANG questionnaire**			
Snoring	63 (27.2)	37 (27.6)	26 (26.5)
Tiredness	123 (53.0)	67 (50.0)	56 (57.1)
Observed apnoea	41 (17.6)	26 (19.4)	15 (15.3)
High blood pressure	106 (45.7)	54 (40.3)	52 (53.1)
BMI > 35 kg/m^2^	0 (0)	0 (0)	0 (0)
Age > 50 years	214 (92.2)	119 (88.8)	95 (96.9)
Neck circumference > 40 cm	68 (29.3)	31 (21.1)	37 (37.6)
Male sex	144 (62.1)	78 (58.2)	66 (67.3)

BMI: body mass index; METS: Metabolic Equivalent of Task Score; ASA-classification: American Society of Anaesthesiologists physical status classification; PVI: pulmonary vein isolation; CAD: coronary artery disease; COPD: chronic obstructive pulmonary disease; OSAS: obstructive sleep apnoea syndrome; SBD: sleep breathing disorder; AHI: apnoea hypopnea index; ODI: oxygen desaturation index; SD: standard deviation.

### 3.2 Outcomes and predictive factors

**Fig. 2:**
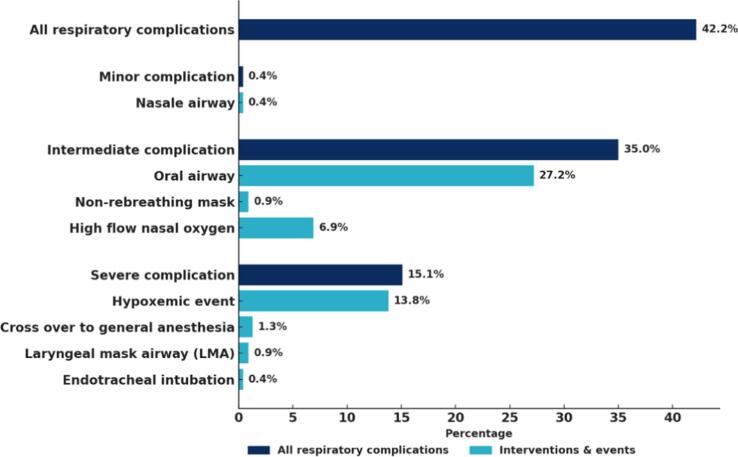
Respiratory complications, with subcategories (minor,intermediate,severe) and interventions in percentages. In several cases more than one respiratory complication ocurred.

The incidence of respiratory complications during CA in patients with AF under PSA was 42.2 %, with 0.4 % classified as minor, 35.0 % as intermediate, and 15.1 % as severe. An oropharyngeal airway (Guedel) was required in 63 patients (27.2 %), whereas a nasopharyngeal airway (Shiley™) was required in 1 patient (0.4 %). A non-rebreathing mask was used in 2 (0.9 %) cases. In 16 (6.9 %) cases high flow oxygen (Optiflow®) was applied. In 3 (1.3 %) cases there was a cross over to general anaesthesia. A laryngeal mask airway (LMA) was placed 2 (0.9 %) times. Endotracheal intubation because of intra-oral bleeding was performed in 1 case (0.4 %). (due to intra-oral blood during the procedure). In 13.8 % of the cases a hypoxic event (saturation < 90 for more than 120 s) occurred. UAC_Desat_ was 42 ± 68 s % (mean ± SD) in a range 4–360. The outlier was 360 s % during the Covid pandemic. This patient had respiratory complaints 2 weeks prior the procedure (not confirmed as a Covid infection). The difference in lowest saturation was 3.9 % (90.1 % ± 4.4 vs 86.2 % ± 6.9). The lowest saturation was not significantly decreased in patients with respiratory complications. There is no significant difference in duration of desaturation. An overview of the respiratory complications is provided in Fig. 2. In several cases more than 1 respiratory complication occurred during PSA.

Supplemental Table 2 shows the results of the univariable logistic regression analysis. The presence of a longer sedation duration was associated with respiratory complications. Also, patient factors as a higher percentage of visceral fat, a higher BMI, an age > 50 years and a neck circumference > 40 cm resulted in a significant increase of respiratory complications. OSAS in the medical history, COPD, apnoea hypopnea index (AHI) and oxygen desaturation index (ODI) did not have a significant effect on respiratory complications. Neither age, activity level, ASA classification or congestive heart failure affected the incidence of respiratory complications. Mean BIS values were similar between cryoballoon and radiofrequency ablation (61.2 ± 14.4 vs. 62.6 ± 12.1; range 28–95 vs. 38–95). In logistic regression analysis, BIS values were not associated with respiratory complications (OR 1.00, 95 % CI 0.98–1.02; *p* = 0.79), whereas RF ablation remained an independent predictor (OR 1.74, 95 % CI 1.02–3.00; *p* = 0.04). Anaesthesia duration was significantly shorter in the cryoballoon ablation group compared with radiofrequency ablation (95.7 ± 20.9 vs. 181.5 ± 40.7 min; mean difference 85.9 min, 95 % CI 77.7–94.0; *p* < 0.001). The incidence of respiratory complications was lower with cryoballoon ablation than with radiofrequency (34.7 % vs. 48.1 %; OR 1.75, 95 % CI 1.02–3.01; *p* = 0.04). Within the radiofrequency group, patients who developed respiratory complications had longer anaesthesia duration compared with those without (188.1 ± 45.9 vs. 175.4 ± 34.5 min; OR 1.01, 95 % CI 1.00–1.02). Supplemental Table 3 shows the univariable logistic regression analyses if we exclude the use of nasal or oral airway as a complication. BMI, visceral fat percentage and duration of sedation remain predictive factors for respiratory complications. Table 2 shows the results of the multivariable logistic regression analysis. The AUROC of the model with all retained variables was 0.72, 95 % CI: 0.65–0.78.

**Table 2:** Multivariable logistic regression analysis.

**Variable**	**OR**	**95 % C.I.**	**p-value**
Tiredness	0.61	0.33–1.09	0.09
Age > 50 years	0.20	0.04–0.70	0.02
Neck circumference > 40 cm	0.65	0.34–1.25	0.19
Visceral fat percentage	1.08	0.99–1.17	0.07
AHI	1.02	0.99–1.05	0.15
OSAS (medical history)	0.24	0.07–0.70	0.01
COPD	0.25	0.05–0.94	0.05
Anaesthesia duration	1.01	1.00–1.01	0.02

All factors with a p-value of < 0,30were used for the multiple logistic regression analyses. The model should be sensitive for factors associated with respiratory complications. AUROC 0.72, 95 % CI: 0.65–0.78.

## 4 Discussion

In this explorative study, the incidence of respiratory complications during CA in patients with AF under PSA was 42.2 %. This high incidence of respiratory complications occurred despite a careful pre-screening protocol at the outpatient clinic. Patients with the clinical diagnosis Obstructive Sleep Apnoea (OSA) prior or high risk based on STOP-Bang questionnaire were excluded for PSA based on the pre-anaesthesia assessment. For the participants with a medical history of obstructive sleep apnoea (OSA) who took part in the study, it was documented that this condition was no longer a current medical issue (e.g., as a result of weight loss or surgical intervention). The relatively low prevalence of previously diagnosed OSA in our study population (9.1 %) likely reflects the exclusion criteria applied during patient selection for PSA in AF ablations. Certain patients with a previously established OSA diagnosis may have been identified through SDB testing prior to the baseline visit. Among patients without previously diagnosed OSA, 67 (28.9 %) were identified with moderate to severe SDB (AHI > 15). Since this prevalence is lower than would be expected in a general AF population, the incidence of respiratory complications would very likely be higher in an unselected AF cohort, and our findings may therefore underestimate the true complication rates. Possibly, the strategies currently used during pre-anaesthesia assessments may work in the general population, but may show some limitations in patients with AF, which may explain the high incidence of respiratory complications in our study. Previous studies have shown, that the STOP-BANG questionnaire is not very accurate in detection SDB in patients with AF [10,25]. Additionally, the VIRTUAL-SAFARI study showed, that SDB is largely underdiagnosed in patients scheduled for AF ablation [12]. As a questionnaire-based pre-selection for SDB screening remains challenging in this population [12]. Interestingly, only 15 (6.5 %) of the patients had severe SDB (AHI > 30 during remote sleep test). The majority of patients with severe SDB (53 % of 15 patients) had respiratory complications. Based on these findings, we continue to consider severe SDB a contraindication for deep sedation during CA for AF. In our experience, SDB screening was frequently incomplete at the time of the pre-anaesthesia evaluation. This suggests a need to revise the current workflow, particularly in high-risk patients. Routine sleep testing prior to the pre-anaesthesia assessment may help optimize patient selection for procedural sedation. Ideally, the anaesthesia assessment should be scheduled only after completion of SDB screening in patients with a high clinical suspicion of SDB.

The incidence of respiratory complications found in our study seems substantially higher than those reported by van Schaik et al., who reported an overall incidence of respiratory complications of 29 % [17]. Both studies used a retrospective observational design and were performed in a tertiary university hospital, with sedation services provided by the Department of Anaesthesiology. There are some important differences between these studies related to procedures and patient selection. For optimal comparison of the data of our study and that of van Schaik [17] it is best to focus on data related to those procedures performed in the heart catheterization room. In the heart catheterization room 1393 procedures were performed under moderate to deep sedation. Then, however, still differences in outcome can be noted: an incidence of complications in our study of 42 % and that reported by van Schaik [17] of 54 %. It needs to be emphasized that the respiratory complications described in the study by van Schaik et al. were all hypoxemic events (defined as a peripheral saturation (SpO_2_) < 90 % lasting for at least two minutes) [17]. On the other hand, in our study the incidence of hypoxemic events was 13.8 %. There are some possible explanations for the observed difference. First, and maybe the most important difference might be sedation procedure related: due to the fact that in our study an oral airway was regularly used (27.6 %) whereas in van Schaik ‘s study this was only very incidentally used (0.1 %). The use of an oral airway is considered to have no negative effects, it is, furthermore, very well accepted by the patient and considered only a minor complication [23]. Another difference, related to patient selection is noted between van Schaik ‘s study and our study, is the BMI-range. In our study BMI was 27.0 ± 3.0 (mean ± SD) with a range 20–33 whereas a BMI of 26.0 ± 5.0 (mean ± SD) with a range 13–60 was reported by van Schaik et al.

The incidence of respiratory complications is high although the risk of potential harm appears low. The clinical effects of short term hypoxemia are unknown. In theory severe hypoxemia can lead to anaerobic metabolism and circulatory changes, resulting in ischemia [16]. A negative impact on neurobehavioral tests 24 h after surgery in patients that showed desaturations on cerebral level is reported [26]. AUC_Desat_ is an end point for assessment of risk of adverse clinical outcomes in clinical sedation studies [24]. All hypoxemic events were mild desaturations with AUC_Desat_ < 150 (seconds %) with the exception of 1 case of moderate desaturation (AUC_Desat_ < 900 s %). Comparison of our data related to the presence of hypoxemic events with other studies is limited because the AUC_Desat_ is often not specifically described. It should furthermore be acknowledged that AUC_Desat_ does not differentiate between short but severe desaturations and prolonged yet mild events, and this implies distinct physiological and clinical consequences. Given the potential for severe hypoxemia to cause harm, even in isolated or transient episodes, it remains essential to implement strategies that prevent desaturation, ensure adequate oxygen delivery, and minimize the risk of long-term adverse outcomes. Further research is warranted to define clinically meaningful thresholds and to better understand the long-term implications of intra-procedural hypoxemia.

The identification of patient-related factors, in particular in view of respiratory complications during moderate or deep sedation, is known to be very limited. We were able to identify several new predictive factors which are associated with the development of per-procedural respiratory complications during CA in patients with AF under PSA. An age > 50 years, the diagnoses OSAS and COPD in the medical history are predictive factors for development of per-procedural respiratory complications. Also neck circumference > 40 cm and visceral fat percentage are predictive factors. The factor tiredness from the STOPBANG questionnaire and AHI are patient characteristics which are closely related with the development of respiratory complications. The duration of sedation is an important predictive procedure related factor of influence. Cryoballoon ablation was associated with significantly shorter anaesthesia duration and a lower incidence of respiratory complications compared with radiofrequency ablation. Depth of sedation as assessed by BIS monitoring, was comparable between the cryoballoon and radiofrequency groups and was not associated with the occurrence of respiratory complications. This suggests that it is not the absolute depth of sedation that drives respiratory risk, but rather the prolonged duration of deep sedation. Accordingly, longer anaesthesia exposure in the radiofrequency group appears to be a key procedural factor contributing to the higher incidence of respiratory complications during PSA. We checked whether the predictive factors remain the same when we would exclude the use of an oral airway as a complication. BMI, visceral fat percentage and duration of sedation remain predictive factors (Supplemental Table 2).

Our study shows that neck circumference and visceral fat percentage are risk factors for respiratory complications. Measuring the neck circumference is an easy measurement that does not require advanced technology. In our study patients reported their neck circumference. Neck circumference and visceral fat percentage are factors closely related to the BMI and this should be taken into account. The latter suggest that these patient characteristics correlate with body composition. It is more likely that patterns of body fat distribution influence the function of the respiratory system via the direct mechanical effect of fat accumulation in the chest and abdominal region.

COPD is recognized as a risk factor for AF. A COPD care pathway can be successfully embedded within an existing AF outpatient clinic infrastructure, using (micro)spirometry and remote analysis of results. Although one in five patients showed results suggestive of an underlying chronic respiratory disease, only 62 % of these patients opted for a referral [27]. From the patient’s perspective this is understandable given the significant overlap in the symptomatology of COPD and AF. However, the latter also implies that a subset of patients undergoing CA for AF may have been undiagnosed and untreated for COPD.

Strengths and Limitations: The strength of our study is the availability of prospective data from the ISOLATION cohort study and ISOLATION ‘light’ registry. In these datasets a major amount of patient factors were registered. Therefore we were able to identify and exclude risk factor for respiratory complications. Especially all factors related to SDB as all patients had remote sleep test for SDB and most of them filled out the STOPBANG questionnaire. By combining data from the ISOLATION baseline characteristics we were able to identify new risk factors. Another strength of our study is the fact that the PSA population of AF patients for CA is basically a rather homogeneous population. Limitations of this study include a selection bias because of the institutional pre-anaesthesia assessment and the retrospective study design. Although the data of prospective data from the ISOLATION cohort study and ISOLATION ‘light’ registry are used, the anaesthesia data are collected retrospectively. An age > 50 years is identified as a risk factor for respiratory complications. However this is 92.2 % of the patients in our population. It might be possible that due to the small group size in the age group < 50 years other factors due to coincidence have been underexposed. Unfortunately the group of participants under 50 years of age is too small to determine a cut-off point at a lower age. Due to the exclusion criteria for PSA during CA a selection bias is part of our study. For instance patients with a BMI > 32 are not included in this study. As a result of the local selection criteria we were unable to determine a BMI-threshold for PSA during CA. The type, dosage, or combination of short-acting sedatives and analgesics used may have influenced the occurrence of respiratory complications. In this observational study, no restrictions were imposed on the choice or administration of these medications. As a result, there was considerable variability in sedation regimens, which reflects the pragmatic, real-world nature of our dataset. Due to the retrospective design of the study the total dose of sedative and analgesic agents is not available. However, continuous infusion was used to minimise the risk of overdose. Furthermore, all sedatives and analgesics were titrated to clinical effect, which makes fixed-dose reporting difficult to interpret due to substantial interindividual variability in pharmacodynamic response. Finally, our multivariable model yielded an area under the receiver operating characteristic curve (AUROC) of 0.72, indicating acceptable—but not strong—predictive accuracy. This moderate performance underscores the limitations of clinical variables alone in capturing the multifactorial risk for respiratory complications during sedation. Future research should improve model performance and support more individualized risk stratification.

Looking forward, it remains to be seen how these findings will apply to emerging ablation technologies, particularly pulsed field ablation (PFA). Especially during single-shot PFA applications direct diaphragmatic or phrenic nerve stimulation may transiently disrupt breathing patterns. In addition, the sensory response to PFA energy appears more intense than that of cryoballoon or radiofrequency ablation, often necessitating deeper sedation levels. These observations are currently anecdotal and beyond the scope of the present study. They emphasise the need for future research into sedation strategies and respiratory safety in the context of PFA.

## 5 Conclusion

First, the incidence of respiratory complications during CA in patients with AF under PSA is 42.2 % and thus relatively high. Second, the following predictive factors were identified for development of per-procedural respiratory complications in these patients: a neck circumference > 40 cm, visceral fat percentage, the factor tiredness from the STOPBANG questionnaire, AHI, the diagnoses OSAS and COPD in the medical history. Although age above 50 years is generally associated with an increased risk of respiratory complications, this threshold demonstrated limited discriminative value in the present cohort, as the vast majority of patients undergoing AF ablation exceeded it. An important procedure related predictive factor is the duration of the PSA. Prospective studies are needed to test, whether a refinement of pre-anaesthesia assessment can help to better identify patients at high risk of experiencing respiratory complications during CA procedures under PSA.

All authors take responsibility for all aspects of the reliability and freedom from bias of the data presented and their discussed interpretation.

## CRediT authorship contribution statement

**Marloes Homberg:** Writing – original draft, Project administration, Methodology, Investigation, Formal analysis, Data curation, Conceptualization. **Konstanze Betz:** Writing – original draft, Investigation, Data curation, Conceptualization. **Sander M.J. van Kuijk:** Writing – review & editing, Methodology, Formal analysis. **Justin Luermans:** Writing – review & editing. **E.A. Joosten:** Writing – original draft, Supervision, Methodology, Conceptualization. **Wolfgang Buhre:** Writing – original draft, Methodology, Conceptualization. **Ulrich Schotten:** Writing – review & editing. **Kevin Vernooy:** Writing – review & editing. **Dominik Linz:** Writing – review & editing, Supervision, Methodology, Conceptualization. **Esther Bouman:** Writing – original draft, Supervision, Methodology, Data curation, Conceptualization.

## Funding

None.

## Declaration of competing interest

The authors declare the following financial interests/personal relationships which may be considered as potential competing interests: Konstanze Betz is social media editor van *International Journal of Cardiology: Heart & Vasculature*.

## References

[1] Garcia R., Waldmann V., Vanduynhoven P., Nesti M., de Oliveira J., Figueiredo M., Narayanan K. (2021). Worldwide sedation strategies for atrial fibrillation ablation: current status and evolution over the last decade. Europace.

[2] Tzeis S., Gerstenfeld E.P., Kalman J., Saad E.B., Sepehri Shamloo A., Andrade J.G. (2024). European heart rhythm association/heart rhythm society/Asia pacific heart rhythm society/Latin American heart rhythm society expert consensus statement on catheter and surgical ablation of atrial fibrillation. Europace.

[3] Hardman B., Karamchandani K. (2023). Management of anesthetic complications outside the operating room. Curr. Opin. Anesthesiol..

[4] Van Gelder I.C., Rienstra M., Bunting K.V., Casado-Arroyo R., Caso V., Crijns H. (2024). 2024 ESC guidelines for the management of atrial fibrillation developed in collaboration with the European Association for Cardio-Thoracic Surgery (EACTS). Eur. Heart J..

[5] Hinkelbein J.J. (2018). European Society of Anaesthesiology and European Board of Anaesthesiology guidelines for procedural sedation and analgesia in adults. Eur. J. Anaesthesiol..

[6] Dobson G., Chong M.A., Chow L., Flexman A., Hurdle H., Kurrek M. (2018). Procedural sedation: a position paper of the Canadian Anesthesiologists' Society. Can. J. Anaesth..

[7] Sigona A., Richman D.C. (2023). Identifying and reducing risks of postoperative pulmonary complications. J. Oral Maxillofacial Anesthesia.

[8] Fernandez-Bustamante A., Frendl G., Sprung J., Kor D.J., Subramaniam B., Martinez Ruiz R. (2017). Postoperative pulmonary complications, early mortality, and hospital stay following noncardiothoracic surgery: a multicenter study by the perioperative research network investigators. JAMA Surg..

[9] Canet J., Gallart L., Gomar C., Paluzie G., Vallès J., Castillo J. (2010). Prediction of postoperative pulmonary complications in a population-based surgical cohort. Anesthesiology.

[10] Linz D., McEvoy R.D., Cowie M.R., Somers V.K., Nattel S., Lévy P. (2018). Associations of obstructive sleep apnea with atrial fibrillation and continuous positive airway pressure treatment: a review. JAMA Cardiol..

[11] Simons S.O., Elliott A., Sastry M., Hendriks J.M., Arzt M., Rienstra M. (2021). Chronic obstructive pulmonary disease and atrial fibrillation: an interdisciplinary perspective. Eur. Heart J..

[12] Betz K., Verhaert D.V.M., Gawalko M., Hermans A.N.L., Habibi Z., Pluymaekers N. (2023). Atrial fibrillation-specific refinement of the STOP-Bang sleep apnoea screening questionnaire: insights from the Virtual-SAFARI study. Clin. Res. Cardiol..

[13] Abumuamar A.M., Dorian P., Newman D., Shapiro C.M. (2018). The STOP-BANG questionnaire shows an insufficient specificity for detecting obstructive sleep apnea in patients with atrial fibrillation. J. Sleep Res..

[14] Pardo E., Bonnet F. (2024). Anaesthesia outside the operating room: a permanent challenge. Curr. Opin. Anaesthesiol..

[15] van Schaik Eva P.C.E. (2021). Hypoxemia during procedural sedation in adult patients: a retrospective observational study. Canadian J. Anesthesia/J. Canadien D'anesthésie..

[16] Bickler P.E., Feiner J.R., Lipnick M.S., Batchelder P., MacLeod D.B., Severinghaus J.W. (2017). Effects of acute, profound hypoxia on healthy humans: implications for safety of tests evaluating pulse oximetry or tissue oximetry performance. Anesth. Analg..

[17] Verhaert D.V.M., Linz D., Chaldoupi S.M., Westra S.W., den Uijl D.W., Philippens S. (2022). Rationale and design of the ISOLATION study: a multicenter prospective cohort study identifying predictors for successful atrial fibrillation ablation in an integrated clinical care and research pathway. Front. Cardiovasc. Med..

[18] Verhaert D.V.M., Betz K., Gawałko M., Hermans A.N.L., Pluymaekers N., van der Velden R.M.J. (2022). A VIRTUAL sleep apnoea management pathway for the work-up of atrial fibrillation patients in a digital remote infrastructure: VIRTUAL-SAFARI. Europace.

[19] Ramsay M.A., Savege T.M., Simpson B.R., Goodwin R. (1974). Controlled sedation with alphaxalone-alphadolone. Br. Med. J..

[20] van Dishoeck A.M., van der Hooft T., Simoons M.L., van der Ent M., Scholte op Reimer W.J. (2009). Reliable assessment of sedation level in routine clinical practice by adding an instruction to the Ramsay Scale. Eur. J. Cardiovasc. Nurs..

[21] Heeger C.H., Bohnen J.E., Popescu S., Meyer-Saraei R., Fink T., Sciacca V. (2021). Experience and procedural efficacy of pulmonary vein isolation using the fourth and second generation cryoballoon: the shorter, the better?. J. Cardiovasc. Electrophysiol..

[22] Heeger C.H., Almorad A., Scherr D., Szegedi N., Seidl S., Baran J. (2025). Temperature-guided high and very high-power short duration ablation for atrial fibrillation treatment: the peQasus multicentre study. Europace.

[23] Roback M.G., Green S.M., Andolfatto G., Leroy P.L., Mason K.P. (2018). Tracking and reporting outcomes of procedural sedation (TROOPS): standardized quality improvement and research tools from the international committee for the advancement of procedural sedation. Br. J. Anaesth..

[24] Niklewski P.J., Phero J.C., Martin J.F., Lisco S.J. (2014). A novel index of hypoxemia for assessment of risk during procedural sedation. Anesth. Analg..

[25] Mehra R., Chung M.K., Olshansky B., Dobrev D., Jackson C.L., Kundel V. (2022). Sleep-disordered breathing and cardiac arrhythmias in adults: mechanistic insights and clinical implications: a scientific statement from the american heart association. Circulation.

[26] Aguirre J.A., Etzensperger F., Brada M., Guzzella S., Saporito A., Blumenthal S. (2019). The beach chair position for shoulder surgery in intravenous general anesthesia and controlled hypotension: impact on cerebral oxygenation, cerebral blood flow and neurobehavioral outcome. J. Clin. Anesth..

[27] van der Velden R.M.J., Hereijgers M.J.M., Arman N., van Middendorp N., Franssen F.M.E., Gawalko M. (2023). Implementation of a screening and management pathway for chronic obstructive pulmonary disease in patients with atrial fibrillation. Europace.

