# Supplementary material for: Peri-procedural respiratory complications in patients undergoing pulmonary vein isolation under procedural sedation and analgesia: Incidence and predictive factors
Source: Int J Cardiol Heart Vasc. 2025 Oct 14;61:101822. doi: 10.1016/j.ijcha.2025.101822 (PMC12550192; doi:10.1016/j.ijcha.2025.101822)
Supplement: Supplementary Data 1 [file mmc1.docx]

**Supplemental Table 1 Additional baseline characteristics of all procedures, without respiratory complications group and with respiratory complications group. Values are number (percentages) unless stated otherwise.**

| **Characteristic** | **Total** | **No complications** | **Respiratory complications** |
| --- | --- | --- | --- |
|  | **n=232** | **n=134** | **n=98** |
| Cerebrovascular disease | 16 (6.9) | 11 (8.2) | 5 (5.1) |
| Peripheral vascular disease | 20 (8.6) | 12 (9.0) | 8 (8.2) |
| Dyslipidaemia | 42 (18.1) | 17 (12.7) | 25 (25.5) |
| Decreased kidney function | 9 (3.9) | 4 (3.0) | 5 (5.1) |
| Smoking status |  |  |  |
| Active | 43 (18.5) | 23 (17.2) | 20 (20.4) |
| Previous <1 year | 1 (0.4) | 1 (0.7) | 0 (0) |
| Previous >1 year | 38 (16.4) | 23 (17.2) | 15 (15.3) |
| Never | 150 (64.7) | 87 (64.9) | 63 (64.3) |
| Alcohol consumption |  |  |  |
| None | 55 (23.7) | 34 (25.4) | 21 (21.4) |
| <5 units/week | 116 (50.0) | 66 (49.3) | 50 (51.0) |
| 5–15 units/week | 50 (21.6) | 27 (20.1) | 23 (23.5) |
| >15 units/week | 11 (4.7) | 7 (5.2) | 4 (4.1) |

**Supplemental Table 2**  Univariable logistic regression analysis.

| Variable |  | OR | 95% CI | P-value |
| --- | --- | --- | --- | --- |
| Sex | Male | 1.48 | 0.86-2.57 | 0.16 |
| Age |  | 1.01 | 0.98-1.04 | 0.50 |
| BMI |  | 1.12 | 1.03-1.23 | 0.01 |
| Activity level |  | 1.49 | 0.46-5.73 | 0.52 |
| ASA classification | ASA2 | 0.68 | 0.03-17.39 | 0.79 |
|  | ASA3 | 1.05 | 0.04-28.22 | 0.97 |
| Atrial fibrillation | Persistent | 1.52 | 0.87-2.67 | 0.14 |
| CAD |  |  |  |  |
| Cerebrovascular disease | TIA | 0.44 | 0.06-1.98 | 0.33 |
|  | Ischemic stroke | 1.03 | 0.20-4.76 | 0.97 |
|  | Haemorrhagic stroke | 6.4e-07 | u.e | 0.99 |
| Congestive heart failure |  | 0.90 | 0.38-2.08 | 0.81 |
| Diabetes mellitus |  | 1.61 | 0.56-4.75 | 0.37 |
| Decreased kidney function |  | 1.75 | 0.45-7.22 | 0.41 |
| COPD |  | 0.49 | 0.10-1.52 | 0.22 |
| OSAS | Medical history | 0.40 | 0.13-1.05 | 0.08 |
| Smoking | Actively | 1.20 | 0.60-2.37 | 0.60 |
|  | Previously (<1 year) | 6.5e-07 | u.e. | 0.99 |
|  | Previously (>1 year) | 9.0e-01 | 0.43-1.85 | 0.78 |
| Alcohol consumption | <5 units/week | 1.23 | 0.64-2.39 | 0.54 |
|  | 5-15 units/week | 1.38 | 0.63-3.02 | 0.42 |
|  | >15 units/week | 0.93 | 0.22-3.45 | 0.91 |
| SBD | AHI | 1.02 | 0.96-1.01 | 0.18 |
|  | ODI | 1.03 | 0.99-1.07 | 0.20 |
|  | desatnadirs | 0.94 | 0.78-1.01 | 0.46 |
|  | Saturation <90% (minutes | 1.03 | 0.88-1.20 | 0.72 |
| STOPBANG | Snoring | 0.95 | 0.52-1.70 | 0.86 |
|  | Tiredness | 1.33 | 0.79-2.26 | 0.28 |
|  | Observed apnoeas | 0.75 | 0.36-1.49 | 0.42 |
|  | High blood pressure | 1.68 | 0.99-2.84 | 0.05 |
|  | BMI>35kg/m2 | - | - | - |
|  | Age>50years | 3.99 | 1.27-17.6 | 0.03 |
|  | Neck circumference >40cm | 2.01 | 1.14-3.59 | 0.02 |
|  | Male | 1.48 | 0.86-2.57 | 0.16 |
| Fatpercentage |  | 1.01 | 0.97-1.04 | 0.71 |
| Muscle percentage |  | 0.98 | 0.92-1.05 | 0.64 |
| Visceral fat percentage |  | 1.11 | 1.04-1.20 | 0.004 |
| Rest metabolism |  | 1.00 | 1.00-1.00 | 0.01 |
| LVEF |  | 1.01 | 0.97-1.05 | 0.61 |
| NTproBNP |  | 1.00 | 0.99-1.01 | 0.78 |
| GFR |  | 1.00 | 0.98-1.02 | 0.92 |
| Anaesthesia duration |  | 1.01 | 0.99-1.00 | 0.006 |

*BMI: body mass index; METS: Metabolic Equivalent of Task Score;* *ASA-classification: American Society of Anaesthesiologists physical status classification; PVI: pulmonary vein isolation; CAD: coronary artery disease; COPD:* *chronic obstructive pulmonary disease; OSAS: obstructive sleep apnoea syndrome; SBD: sleep breathing disorder; AHI: apnoea-hypopnea index; ODI: oxygen desaturation index; LVEF: left ventricular ejection fraction; GFR: glomerular filtration rate; OR: Odds Ratio; SD: standard deviation*; *u.e: unable to estimate*.

**Supplemental Table 3** Per-procedural respiratory complications were defined if a non-rebreathing mask or high flow oxygen was indicated, or a hypoxemic event (a peripheral saturation <90% lasting for at least 120 seconds) or a conversion to general anaesthesia with laryngeal mask airway (LMA) or tracheal intubation.

| **Variable** |  | **OR** | **95% CI** | **P-value** |
| --- | --- | --- | --- | --- |
| **Sex** | Male | 1.78 | 0.90-3.71 | 0.11 |
| **Age** |  | 1.01 | 0.98-1.05 | 0.44 |
| **BMI** |  | 1.16 | 1.04-1.31 | 0.009 |
| **Activity level** |  | u.e | u.e | 0.99 |
| **ASA classification** | ASA2 | u.e | u.e | 0.99 |
|  | ASA3 | u.e | u.e | 0.99 |
| **Atrial fibrillation** | Persistent | 1.03 | 0.51-2.01 | 0.94 |
| **CAD** |  | 1.45 | 0.45-4.04 | 0.50 |
| **Cerebrovascular disease** | TIA | 1.33 | 0.19-5.98 | 0.74 |
|  | Ischemic stroke | 0.65 | 0.03-3.93 | 0.69 |
|  | Haemorrhagic stroke | u.e | u.e | 0.99 |
| **Congestive heart failure** |  | 1.28 | 0.44-3.24 | 0.62 |
| **Diabetes mellitus** |  | 0.59 | 0.09-2.23 | 0.50 |
| **Decreased kidney function** |  | 1.13 | 0.16-4.87 | 0.88 |
| **COPD** |  | 0.34 | 0.02-1.84 | 0.31 |
| **OSAS** | Medical history | u.e | u.e. | 0.99 |
| **Smoking** | Actively | 1.76 | 0.78-3.82 | 0.16 |
|  | Previously (<1 year) | u.e | u.e. | 0.99 |
|  | Previously (>1 year) | 1.21 | 0.48-2.85 | 0.67 |
| **Alcohol consumption** | <5 units/week | 2.08 | 0.88-5.50 | 0.11 |
|  | 5-15 units/week | 2.17 | 0.79-6.32 | 0.14 |
|  | >15 units/week | 0.69 | 0.03-4.50 | 0.74 |
| **SBD** | AHI | 1.00 | 0.97-1.03 | 0.96 |
|  | ODI | 1.00 | 0.94-1.05 | 0.91 |
|  | desatnadirs | 0.76 | 0.60-0.97 | 0.02 |
|  | Saturation <90% (minutes) | 1.05 | 0.87-1.22 | 0.53 |
| **STOPBANG** | Snoring | 0.67 | 0.30-1.40 | 0.31 |
|  | Tiredness | 0.91 | 0.48-1.73 | 0.76 |
|  | Observed apnoeas | 0.78 | 0.30-1.79 | 0.58 |
|  | High blood pressure | 1.81 | 0.95-3.50 | 0.07 |
|  | BMI>35kg/m2 | - | - | - |
|  | Age>50years | u.e. | u.e. | 0.99 |
|  | Neck circumference >40cm | 1.88 | 0.96-3.66 | 0.06 |
|  | Male | 1.78 | 0.90-3.71 | 0.11 |
| **Fatpercentage** |  | 1.02 | 0.96-1.06 | 0.44 |
| **Muscle percentage** |  | 0.96 | 0.89-1.04 | 0.35 |
| **Visceral fat percentage** |  | 1.16 | 1.07-1.28 | 0.0006 |
| **Rest metabolism** |  | 1.00 | 1.00-1.00 | 0.06 |
| **LVEF** |  | 1.00 | 0.96-1.05 | 0.96 |
| **NTproBNP** |  | 1.00 | 0.99-1.01 | 0.71 |
| **GFR** |  | 1.01 | 0.98-1.04 | 0.48 |
| **Anaesthesia duration** |  | 1.01 | 0.99-1.00 | 0.05 |

*BMI: body mass index; METS: Metabolic Equivalent of Task Score;* *ASA-classification: American Society of Anaesthesiologists physical status classification; PVI: pulmonary vein isolation; CAD: coronary artery disease; COPD:* *chronic obstructive pulmonary disease; OSAS: obstructive sleep apnoea syndrome; SBD: sleep breathing disorder; AHI: apnoea-hypopnea index; ODI: oxygen desaturation index; LVEF: left ventricular ejection fraction; GFR: glomerular filtration rate; OR: Odds Ratio; SD: standard deviation; u.e: unable to estimate.*
